# Personality, negative affectivity and emotional intelligence: gender-differentiated relationships with physical exercise

**DOI:** 10.3389/fpsyg.2023.1293310

**Published:** 2023-11-30

**Authors:** Yolanda Campos-Uscanga, Vianey Argüelles-Nava, Aurora Mejia-Castillo, Rosa Maribel Barradas-Landa, Kassandra Rosas-Campos, Mirei Narave-Moreno

**Affiliations:** ^1^Institute of Public Health, Universidad Veracruzana, Xalapa, Veracruz, Mexico; ^2^Faculty of Psychology, Universidad Veracruzana, Xalapa, Veracruz, Mexico; ^3^Directorate of Sports Activities, Universidad Veracruzana, Xalapa, Veracruz, Mexico; ^4^Faculty of Medicine, Universidad Veracruzana, Xalapa, Veracruz, Mexico

**Keywords:** personality traits, emotional intelligence, negative affect, physical activity, women

## Abstract

**Introduction:**

Physical exercise is one of the most relevant lifestyle choices for the prevention of diseases; however, participation in this type of activity remains low. Therefore, it is necessary to deepen the understanding of related psychological factors in men and women.

**Objective:**

To determine whether personality traits, emotional intelligence and negative affective are differentially related to physical exercise characteristics in men and women.

**Method:**

A cross-sectional study was conducted with 579 physically active people (61.1% men) between 18 and 59 years of age. The Big Five Inventory (BFI-15p), Brief Emotional Intelligence Scale (EQ-i-M20), and Depression, Anxiety and Stress Scale (DASS-21) were used.

**Results:**

Compared with men, women exercised fewer days and minutes per week, had fewer years of participation and performed fewer different physical exercises. On the emotional intelligence scale, compared with men, women showed less stress management, adaptability and general mood but greater interpersonal intelligence. With regard to personality traits, compared with men, women showed greater openness, conscientiousness, agreeableness, and neuroticism. In males, heightened levels of general mood and extraversion were associated to a lasting commitment to physical exercise over time. Conversely, in females, depression was negatively associated with the years dedicated to physical exercise.

**Conclusion:**

Distinct approaches are essential for men and women, acknowledging the varied ways psychological factors are linked to physical exercise based on gender.

## 1 Introduction

Given the high prevalence of chronic noncommunicable diseases, it is increasingly important to opt for healthy lifestyles. Among these, physical activity emerges as a crucial element in disease prevention and the enhancement of mental health. Nevertheless, participation in physical exercise or sports remains limited in the Mexican population, with a prevalence of 49.5% for men and 35.6% for women (National Institute of Statistics and Geography. Instituto Nacional de Estadística y Geografía, 2023).

Physical exercise is inversely related to psychological stress (Fontana et al., 2022), and this relationship may be bidirectional: participation in physical activity is related to less stress/negative affect during the subsequent hours of the day, and feeling less stressed at the beginning of the day translates into greater physical activity (Schultchen et al., 2019). In a similar way, physical exercise improves anxiety symptoms in young adults without major disorders (Gordon et al., 2020), and the increase in depression scores is related to the lack of physical exercise, while moderate- and high-intensity training decreases scores (Paolucci et al., 2018).

The associations of physical exercise with the emotional state are relatively well known; however, it has recently been shown that participation in physical exercise of any type and of moderate to vigorous intensity is positively related to emotional intelligence in men and women (Skurvydas et al., 2022). The term emotional intelligence is utilized to denote the cognitive processes engaged in recognizing, utilizing, comprehending, and managing one’s own and others’ emotional states for the purposes of problem-solving and behavior regulation (Mayer and Salovey, 1997). Therefore, emotional intelligence has been included in studies from the educational field related to physical education, competitive sports and sport psychology (Ubago-Jiménez et al., 2019), and even other lifestyles.

The evidence shows higher levels of emotional intelligence and physical activity in free time among men than among women (Acebes-Sánchez et al., 2019); therefore; the study of both phenomena deserves an analysis by sex.

Another aspect of subjectivity that has been linked to physical exercise is personality traits. Less neuroticism is related to a higher frequency of physical exercise (Kroencke et al., 2019; Kekäläinen et al., 2020; Gacek et al., 2021) and higher sports performance (Stine et al., 2019). Greater extraversion is related to a greater frequency of physical exercise (Kroencke et al., 2019; Kekäläinen et al., 2020; McDowell et al., 2020; Gacek et al., 2021; Okely et al., 2021). Similarly, awareness and agreeableness tend to improve performance in athletes (Stine et al., 2019), but in young people, openness and agreeableness are negatively related to the probability being physically active (Bessey, 2018; Hearon and Harrison, 2021), and athletes have significantly higher consciousness sores than do people who do not engage in physical exercise (Michałowska-Sawczyn et al., 2019; Humińska-Lisowska et al., 2022). Finally, elite athletes show greater openness than do average athletes (Mitić et al., 2021), and openness has a moderating effect between exercise intention and exercise behavior (Ma et al., 2022).

The previous findings show relationships between physical exercise and personality traits, but most of the studies include athletes in a specific sport or specific population groups; therefore, data for the general population are limited. Additionally, some studies have found clear and consistent associations regarding personality traits, while others present divergent results. For example, extraversion has been identified as a risk factor, without confirmation through other studies; apparently, the differences are attributed to gender (Sutin and Terracciano, 2017).

It is necessary to investigate potential gender differences in the relationships between the duration of physical exercise and the psychological factors examined in this study (personality traits, negative affectivity, and emotional intelligence) and to understand the underlying reasons for women’s lower adherence to physical exercise. This knowledge will also prove beneficial for the male population because it will enable the enhancement of strategies to promote adherence to physical activity by comprehending the connections with certain psychological factors.

In the realm of sports and exercise psychology, collating research results to promote involvement in physical activities is a pivotal area of interest. Information regarding the psychological elements that are linked to prolonged engagement in physical activity will be beneficial for professionals and academics within this field. Nonetheless, there is an absence of studies on the subject.

Therefore, the objective of this work was to determine whether personality traits, emotional intelligence and negative affective state are related to the characteristics of physical exercise in men and women. We propose the following hypotheses: (1) females tend to indicate higher levels of negative emotions and neuroticism and lower emotional intelligence than males; (2) negative affectivity and neuroticism have an inverse correlation with the duration of engagement in physical activity; (3) emotional intelligence and extraversion exhibit a positive association with the duration of engagement in physical activity; and (4) the associations between years of physical exercise, personality traits, negative emotions, and emotional intelligence may vary between males and females.

## 2 Method

### 2.1 Design

A cross-sectional study was conducted for analytical purposes.

### 2.2 Sample

Out of 600 individuals invited to participate in the exercise study, 21 declined, resulting in a final sample of 579 physically active people (61.1% men). Nonprobabilistic convenience sampling was carried out. People between 18 and 59 years of age who, at the time of the interview, performed some type of physical exercise, regardless of experience, were included. People with physical or cognitive limitations that prevented them from answering the questionnaires autonomously were excluded.

### 2.3 Instruments

A questionnaire was applied to collect data such as age, sex, and physical activity characteristics (type, diversity, frequency, duration and places where practiced); later, scales were applied to measure personality traits, emotional state and emotional intelligence.

The short version of the Big Five Inventory (BFI-15p) comprises 15 items that assess five dimensions of personality traits: extraversion, neuroticism, conscientiousness, openness and agreeableness. It is scored using a Likert-type scale with 5 response options ranging from “strongly agree” to “strongly disagree.” The instrument was validated in the Mexican population through exploratory structural equation models and presented satisfactory fit indices, coherent internal structure and adequate reliability coefficients, demonstrating adequate psychometric properties for its use in this population (Dominguez-Lara et al., 2022).

The brief emotional intelligence scale (EQ-i-M20), which consists of 20 items scored using a Likert-type scale, was used to measure 5 factors: intrapersonal emotional intelligence, interpersonal emotional intelligence, stress management, adaptability and general mood. Each item has four response options ranging from “it never happens to me” to “it always happens to me.” For the stress management subscale, the higher the score is, the lower the stress management; for the rest of the subscales, the higher the scores are, the higher the measured factor. In the Mexican population, the initial 5-factor structure demonstrated favorable fit indices and reliability coefficients (Dominguez-Lara and Campos-Uscanga, 2021).

The Depression, Anxiety and Stress Scale (DASS-21) was used to measure the negative emotional state. It has been validated in the Mexican population and is composed of 21 items with three subscales that measure stress, anxiety, and depression. The responses are scored using a Likert-type scale ranging from 1 to 4 points (“never” to “almost always”), considering a time period of the last month (Salinas-Rodríguez et al., 2023).

### 2.4 Procedures

During the months of June and July 2022, public and private spaces for physical exercise in the city of Xalapa, Veracruz, Mexico, were visited at different times. Participants were encouraged to engage voluntarily by responding to the printed questionnaire. Approximately 15 min were allocated to complete it. Subsequently, the interviewer ensured that all questions were answered.

### 2.5 Data analysis

The analyses were carried out using SPSS version 21. Participant characteristics were contrasted by sex: quantitative variables through the t test for independent samples and qualitative variables by the chi-square test. The magnitude of the disparities between the groups was assessed using Cohen’s d test, where a value of 0.2 indicates a small effect size, 0.5 indicates a moderate effect size, and 0.8 indicates a large effect size (Cohen, 1988). All association analyses were performed independently for men and women. The study utilized Pearson’s coefficient of determination for bivariate analysis. In the multivariate linear regression (forward stepwise selection), three models were employed for each gender, with years of physical exercise as the dependent variable. The first model focused on personality traits, incorporating all personality traits as independent variables. The second model centered on negative affectivity, including all dimensions of negative affectivity as independent variables. The third model was based on emotional intelligence, with all dimensions of emotional intelligence introduced as independent variables. In every model, age, frequency (number of days) of physical exercise, and duration (minutes per week) of physical exercise were included as confounding variables.

### 2.6 Ethical considerations

The protocol was approved by the Research Committee and the Research Ethics Committee of the Public Health Institute of Veracruzana University under registration numbers CEI-ISP-09-2022 and CEI-ISP-UV-R11/2022, respectively.

## 3 Results

Compared with men, there was a higher proportion of women with children, with a bachelor’s degree or higher, with a stable partner and who exercise for health. Compared with women, men largely stated that they exercised by participating in competitions and that their reason for exercising was to participate in competitions (Table 1).

**Table 1:** Comparison of sociodemographic characteristics and physical exercise by sex.

Variable	Men (*n* = 354) %	Women (*n* = 225) %	X^2^	*p*
Has children	15.6	24.9	7.36	0.007
Bachelor’s degree or higher	40.0	55.9	13.74	<0.001
Has a stable partner	14.2	21.4	4.84	0.028
Exercises with others	83.1	68.9	15.68	<0.001
Participate in competitions	58.0	27.0	52.47	<0.001
Reasons to exercise				
Health	71.9	87.9	5.62	0.018
Recreation	51.0	45.1	0.57	0.450
Competition	37.7	14.8	34.89	<0.001
Aesthetic	37.9	42.9	1.41	0.236

Compared with men, women were older, exercised fewer days a week, exercised fewer minutes a week, had fewer years of experience exercising and engaged in fewer different physical exercises. Regarding emotional intelligence, women had lower scores for adaptability but higher scores for interpersonal and stress management (higher scores indicate poorer stress management). Regarding personality traits, women had higher openness, conscientiousness, agreeableness, and neuroticism scores (Table 2).

**Table 2:** Comparison of means of physical exercise characteristics, emotional intelligence, negative affectivity and personality traits by sex.

VARIABLE	Men (*n* = 354)		Women (*n* = 225)		*p*	*Cohen’s d*
	Medium	SD	Medium	SD		
Age	25.38	8.95	27.41	9.70	0.012	−0.22
Days per week	4.82	1.29	4.57	1.23	0.023	0.20
Minutes per week	757.23	497.30	585.15	446.26	<0.001	0.36
Number of exercises	1.69	0.80	1.37	0.62	<0.001	0.44
Years of practice	7.57	8.79	3.18	5.27	<0.001	0.58
Emotional intelligence						
Intrapersonal	10.45	2.88	10.20	2.95	0.324	0.09
Interpersonal	11.32	2.20	11.70	2.11	0.042	−0.18
Stress management	7.88	2.51	8.81	2.77	<0.001	−0.36
Adaptability	11.74	2.24	11.29	2.13	0.018	0.20
General mood	12.29	2.68	11.87	2.86	0.072	0.15
Negative affectivity						
Stress	12.75	4.36	13.27	4.70	0.179	−0.12
Anxiety	12.75	3.38	13.11	3.97	0.267	−0.10
Depression	12.44	3.93	12.07	4.07	0.271	0.09
Personality traits						
Openness	11.47	2.38	11.95	2.41	0.019	−0.20
Neuroticism	8.79	2.55	9.90	2.86	<0.001	−0.42
Conscientiousness	11.10	2.40	11.94	2.23	<0.001	−0.36
Extraversion	10.90	2.75	10.76	2.87	0.545	0.05
Agreeableness	11.22	2.27	11.80	2.20	0.003	−0.26

Days of exercise per week were negatively associated with anxiety in men and positively associated with neuroticism in women. Minutes of exercise per week were positively associated with neuroticism in both genders, positively associated with stress and anxiety, and negatively associated with intrapersonal emotional intelligence in women. The number of different exercises performed was positively associated with adaptability and openness in women. Finally, years of physical exercise was negatively associated with depression, stress and neuroticism in both groups and positively related to intrapersonal emotional intelligence, adaptability, general mood, conscientiousness and extraversion in men (Table 3).

**Table 3:** Correlations of the characteristics of physical exercise with emotional intelligence, negative affectivity and personality traits by sex.

	Days per week	Minutes per week	Number of exercises	Years of practice
*Intrapersonal emotional intelligence*				
Men	−0.002	0.018	0.027	0.153^**^
Women	−0.085	−0.162^*^	0.118	0.059
*Interpersonal emotional intelligence*				
Men	0.100	−0.006	−0.003	0.006
Women	−0.016	0.049	0.045	0.022
*Emotional intelligence, stress management*				
Men	−0.018	0.053	−0.019	−0.009
Women	0.046	0.061	0.023	−0.129
*Emotional intelligence, adaptability*				
Men	0.056	0.070	0.077	0.133^*^
Women	0.016	0.042	0.141*	0.022
*Emotional intelligence, general mood*				
Men	0.089	0.049	0.080	0.212^**^
Women	−0.007	−0.120	0.074	0.123
*Stress*				
Men	−0.067	0.080	−0.062	−0.115^*^
Women	0.098	0.169^*^	−0.030	−0.166^*^
*Anxiety*				
Men	−0.117^*^	0.002	−0.0056	−0.059
Women	0.053	0.134^*^	−0.031	−0.078
*Depression*				
Men	−0.075	0.026	−0.095	−0.130^*^
Women	0.043	0.095	−0.100	−0.165^*^
*Openness*				
Men	0.010	0.001	0.049	0.093
Women	0.014	−0.007	0.138*	−0.029
*Neuroticism*				
Men	−0.063	0.155^**^	0.017	−0.141^**^
Women	0.157^*^	0.136^*^	−0.072	−0.207^**^
*Conscientiousness*				
Men	−0.005	−0.067	0.052	0.244^**^
Women	−0.031	−0.125	0.017	−0.025
*Extraversion*				
Men	0.084	0.025	0.082	0.223^**^
Women	0.006	−0.100	0.051	0.111
*Agreeableness*				
Men	0.006	0.050	0.035	0.089
Women	−0.080	0.013	−0.003	0.034

^*^p < 0.05, ^**^p < 0.01.

**Figure 1:**
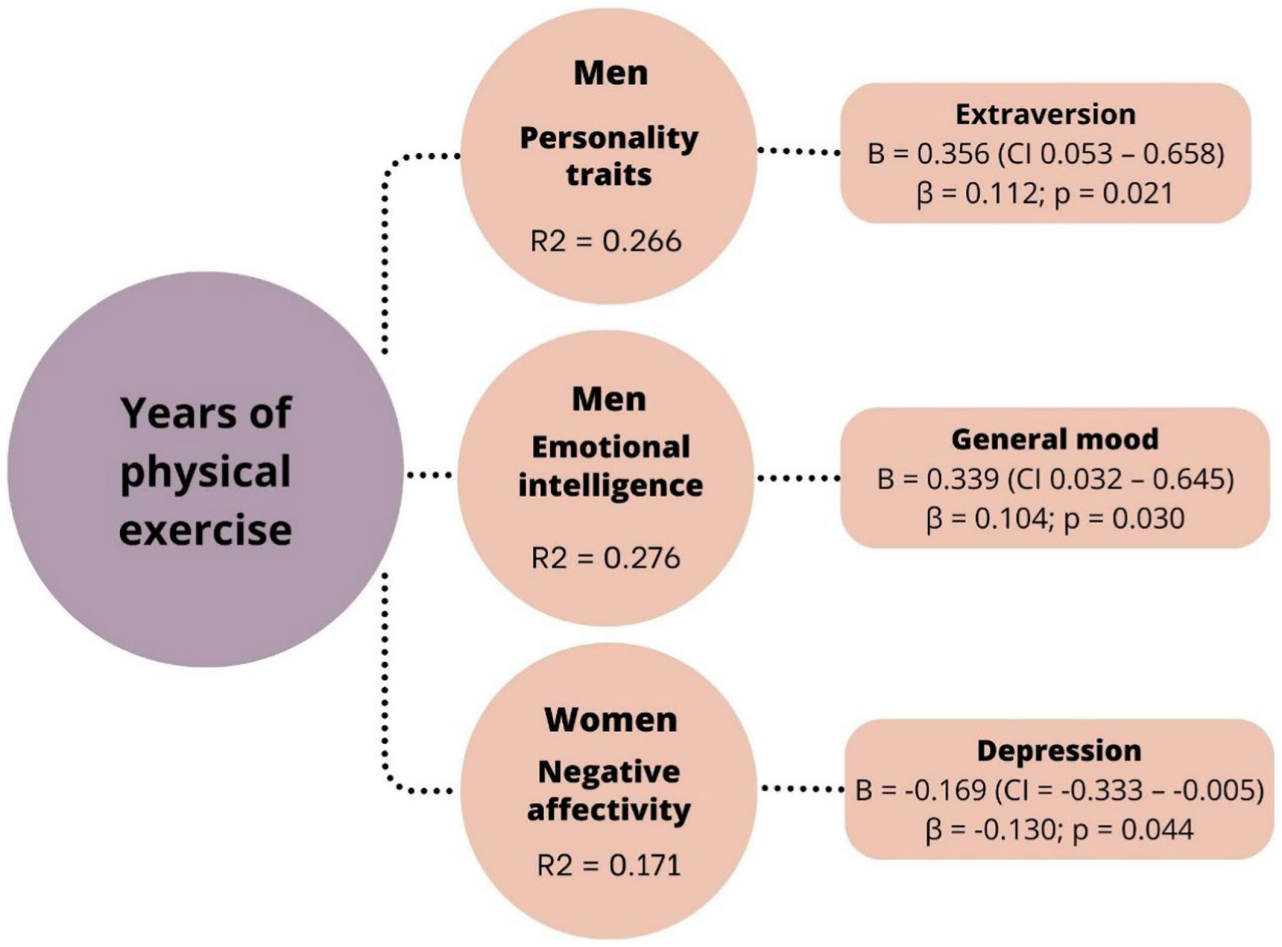
Multivariate models for years of physical exercise (dependent variable) by sex. B, Unstandardized coefficients; CI, Confidence interval; β, Standardized coefficients.

In males, heightened levels of general mood and extraversion were associated to a lasting commitment to physical exercise over time. Conversely, in females, depression was negatively associated with the years dedicated to physical exercise (Figure 1).

## 4 Discussion

The challenges related to increasing physical activity among populations are great due to the wide range of environmental and social factors that are associated with such activity; however, personal and subjective factors may also contribute to low rates of physical exercise, a problem that is more accentuated among women (Instituto Nacional de Estadística y Geografía, 2023). These data are consistent with our findings. The frequency, duration, variety of physical exercises and years of practice were lower among women than men. This underscores the significance of emphasizing the promotion of physical exercise among women.

Additionally, in agreement with previous studies (Acebes-Sánchez et al., 2019), regarding emotional intelligence, men had better adaptability and stress management scores than women, and women had higher interpersonal intelligence scores than men. These findings seem to indicate that women focus on the well-being of others.

However, women reported exercising for health more often than men, suggesting an intention of self-care, an aspect that provides an area of opportunity for the development of interventions. While the majority of men exercise for health and recreation purposes, many exercise to participate in competitions. The motivations for exercising are different between men and women; this insight suggests that strategies for promoting physical exercise need to be rethought.

Regarding personality traits, the scores for desirable traits such as openness, conscientiousness, and agreeableness were higher for women than for men, as was the score for neuroticism, which is associated with worse sports performance (Stine et al., 2019) and a lower frequency of physical exercise (Gacek et al., 2021).

Based on the obtained results, we can partially support hypothesis one, suggesting that women tend to report higher levels of negative affect and neuroticism while exhibiting lower emotional intelligence than men. This conclusion is drawn from the fact that although women scored higher in one aspect of emotional intelligence, men scored higher in two dimensions. Similarly, women scored higher in neuroticism as anticipated, but no significant differences were noted in negative affectivity.

As per Kekäläinen et al. (2020), neuroticism displayed a negative correlation with physical activity in women. Notably, in the current study, the preliminary analysis suggested a negative link between neuroticism and the total duration of exercise over the years. However, it also unveiled a positive association between neuroticism and exercise frequency, encompassing both the number of days and minutes per week. Nevertheless, in the multivariate analysis, the connection between neuroticism and the duration of physical exercise over the years was not sustained.

Similarly, in the bivariate analysis, stress showed a positive correlation with more minutes of training per week and a negative correlation with the overall duration of exercise over the years. These patterns may suggest exercise habits that women adopt initially but are not sustainable over time. However, the multivariate analysis did not confirm a relationship between the duration of physical exercise over the years and stress.

In women, multivariate models considering personality traits and emotional intelligence showed no associated variables. Only in the model involving negative affectivity was depression identified as an associated variable, consistent with a prior study (Paolucci et al., 2018). The modifiability of depressive symptoms adds significance to these findings for early detection and intervention formulation. Interventions based on these results can empower women to foster healthier emotional states, increasing the likelihood of adhering to physical exercise and enhancing overall well-being.

Through multivariate analysis, it was established that, in men, the general mood dimension of emotional intelligence and the personality trait of extraversion were positively associated with a greater number of years devoted to physical training. These factors serve as positive indicators linked to physical activity, aligning with findings from other studies (Stine et al., 2019; Gacek et al., 2021). Given the relatively stable nature of personality traits and emotional intelligence, these specific characteristics may play a crucial role in motivating men’s engagement in physical exercise; hence, they could potentially serve as predictors for success in adhering to physical exercise.

Extraversion and general mood favor permanence in participation in physical exercise among men but not among women, indicates the need for different approaches to promote exercise. There were no differences in the scores for these variables between the sexes, implying that differences in physical activity experiences are not explained *per se* by scores but by different ways in which the variables are related to physical exercise based on sex.

Based on the aforementioned findings, hypotheses two, three, and four are partially accepted. It was observed that only a limited number of dimensions within personality traits, emotional intelligence, and negative affectivity play a role in explaining the duration of engagement in physical exercise, and this relationship varies depending on gender. In women, depression was recognized as a potential risk factor, whereas attributes such as extraversion and general mood were identified as protective factors for men.

A limitation of this work is that the analyses carried out do not allow the establishment of causal associations. However, it can be assumed that personality traits preceded the other traits. In the future, longitudinal studies that allow a better analysis of the influence of the different variables involved in physical exercise are warranted.

The most noteworthy outcome of this study lies in the capacity to discern the specific ways in which psychological factors and the continuity of engagement in physical exercise vary between men and women. This understanding sheds light on the heightened hurdles women face in terms of participation and sustained commitment, which are potentially linked to negative affectivity. Consequently, these findings are relevant for crafting interventions tailored to each gender to bolster adherence to physical exercise regimens.

To evaluate the efficacy of interventions meant to lessen negative emotionality and its effect on physical activity, longitudinal studies, which may even employ experimental methods, are necessary to further our understanding of these mechanisms.

## 5 Conclusion

Compared to men, women obtained lower scores on psychological factors considered risk factors, such as neuroticism and stress management. Women achieved higher scores on certain factors that are considered protective, such as interpersonal intelligence, openness, conscientiousness, and agreeableness.

In men, extended periods of physical exercise correlate with elevated levels of general mood and extraversion. Conversely, for women, the duration of physical exercise is negatively influenced by depression. Men’s enduring commitment to physical exercise is fortified by persistent attributes such as personality traits and emotional intelligence. In contrast, women face constraints on the continuity of long-term physical exercise due to malleable factors like negative affectivity.

These findings indicate that because there are distinct relationships between the variables based on gender, strategies aimed at encouraging longer durations of physical exercise should be tailored to each sex.

## Data availability statement

The original contributions presented in the study are included in the article/supplementary materials, further inquiries can be directed to the corresponding author.

## Ethics statement

The studies involving humans were approved by Research Ethics Committee of the Public Health Institute of Veracruzana University. The studies were conducted in accordance with the local legislation and institutional requirements. The participants provided their written informed consent to participate in this study.

## Author contributions

YC-U: Conceptualization, Data curation, Formal analysis, Investigation, Methodology, Project administration, Resources, Software, Supervision, Validation, Visualization, Writing – original draft, Writing – review & editing. VA-N: Data curation, Investigation, Visualization, Writing – review & editing. AM-C: Validation, Writing – review & editing. RB-L: Investigation, Resources, Writing – review & editing. KR-C: Investigation, Validation, Writing – review & editing. MN-M: Validation, Writing – review & editing.
